# The Role of the Apelin/APJ System in the Regulation of Liver Disease

**DOI:** 10.3389/fphar.2017.00221

**Published:** 2017-04-24

**Authors:** Xinrui Lv, Jing Kong, Wei-Dong Chen, Yan-Dong Wang

**Affiliations:** ^1^Key Laboratory of Receptors-Mediated Gene Regulation and Drug Discovery, School of Medicine, Henan UniversityKaifeng, China; ^2^Key Laboratory of Molecular Pathology, School of Basic Medical Science, Inner Mongolia Medical UniversityHohhot, China; ^3^State Key Laboratory of Chemical Resource Engineering, College of Life Science and Technology, Beijing University of Chemical TechnologyBeijing, China

**Keywords:** Apelin, APJ, liver disease, peptide, angiotensin II receptor like-1

## Abstract

Apelin is an endogenous peptide that is a ligand for the APJ receptor (angiotensin II receptor like-1, AT-1). The apelin/APJ system is distributed in diverse periphery organ tissues. It has been shown that the apelin/APJ system plays various roles in physiology and pathophysiology of many organs. It regulates cardiovascular development or cardiac disease, glycometabolism and fat metabolism as well as metabolic disease. The apelin/APJ system participates in various cell activities such as proliferation, migration, apoptosis or inflammation. However, apelin/APJ function in the liver is still under investigation. In the liver, the apelin-APJ system could play an inhibitory role in liver regeneration and promote Fas-induced apoptosis. It may participate in the formation of hepatic fibrosis or cirrhosis, and even cancer. In this review, we summarize the role of the apelin/APJ system in liver disease.

## Introduction

As an endogenous peptide, apelin acts as a ligand for the APJ receptor (angiotensin II receptor like-1, AT-1). Apelin is a 77 amino acid preproprotein and its isoforms include apelin-13, -16, -17 and -36. Each isoform has distinct activity and the shorter isoform is a more potent activator for APJ. Both apelin-13 and apelin-17 have much stronger activity than apelin-36 (Kawamata et al., 2001; De Mota et al., 2004; Chaves-Almagro et al., 2015). The receptor binding affinities of apelin-13 and apelin-36 are different, which causes different intracellular signaling of APJ. APJ is composed of 377 amino acids. It has a seven transmembrane domain and is a G protein-coupled receptor. It is believed that apelin is the only endogenous ligand for APJ (Simpkin et al., 2007; Lee et al., 2010; Chapman et al., 2014).

It has been demonstrated that the apelin/APJ system plays important and various roles in the physiology and pathophysiology of many organs, including regulation of blood pressure, cardiac contractility, angiogenesis, metabolic balance, and cell proliferation, apoptosis or inflammation. However, whether apelin/APJ has important functions in liver disease is still under investigation. Liver disease includes liver fibrosis or cirrhosis, viral hepatitis, hepatocellular carcinoma (HCC), alcoholic fatty liver (ALD) as well as non-alcoholic fatty liver disease (NALD). In this review, we summarize the latest studies on the functions of the apelin/APJ system in liver disease.

## Distribution and Expression of Apelin in Liver

Apelin is expressed in the heart, endothelium, vascular smooth muscle cells (VSMCs), brain, kidney, testis, ovary, liver and adipose tissue, with the highest expression levels in the lung and the mammary gland (Hosoya et al., 2000; Kawamata et al., 2001). Chu et al. (2013) reported that APJ is expressed in primary hepatocytes, liver tissues of mouse and HepG2 cells, but the expression levels of apelin were lower than that of APJ in liver. The study of Jeong et al. (2014) showed that apelin expression was decreased by silencing the activating transcription factor 4 (ATF4) in HepG2 cells. Principe et al. (2008) reported that the apelin/APJ expression was increased sharply in the hepatic tissue of cirrhotic rats, and APJ expression was 300 times more than that of the control groups. In comparison with the control, the circulating levels of apelin were markedly increased in rats with cirrhosis. It has been found that APJ was expressed in Kupffer cells, and it inhibited liver regeneration after partial hepatectomy in mice (Yoshiya et al., 2015). In addition, the apelin/APJ was expressed in human hepatic stellate cells (HSCs), and promoted liver fibrosis or cirrhosis progression (Melgar-Lesmes et al., 2010; Yokomori et al., 2011).

## The Downstream and Upstream Factors of Apelin/APJ

**FIGURE 1:**
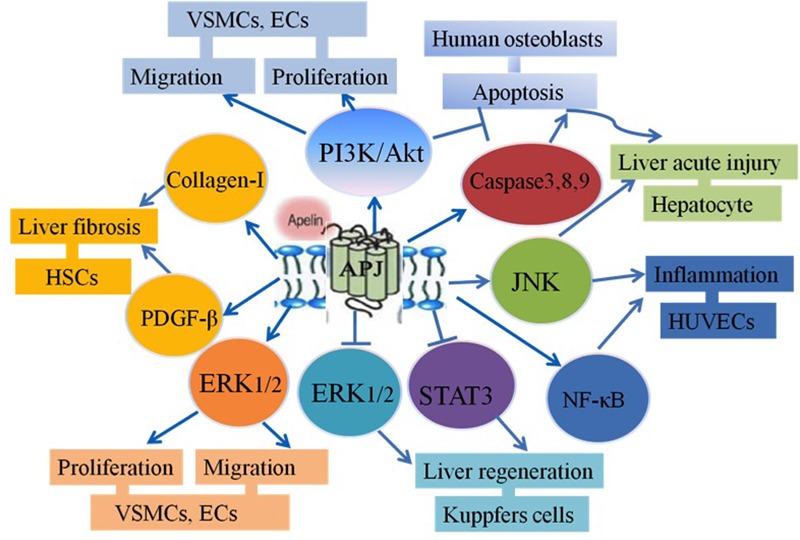
**The functions of apelin/APJ in liver disease**.

It has been shown that apelin/APJ signaling is coupled to pertussis toxin (PTX)-sensitive G proteins and activates protein kinase C (PKC) in cultured cells (Masri et al., 2002) or mouse tissues (Liu et al., 2015). Both apelin-36 and apelin-13 can activate the same intracellular effectors. However, there are differences in their desensitization patterns and coupled G-proteins (Masri et al., 2006). The studies have demonstrated that apelin binds to APJ, which leads to the phosphorylation of protein kinase B (Akt) and extracellular signal-regulated kinase (ERK) in many cell proliferation or migration activities. The Akt signaling pathway has been shown to contribute to cell migration. Also, the ERK/Akt-p70S6K pathway regulates cell proliferation (Masri et al., 2004; Liu et al., 2010; Langelaan et al., 2013). It has been recently shown that a heterodimer is formed with APJ and K-opioid receptors and this leads to the phosphorylation of ERK, resulting in increasing cell proliferation (Li et al., 2012; Chaves-Almagro et al., 2015). Moreover, APJ/apelin system induces intercellular adhesion molecule-1 (ICAM-1) expression via the NF-κB/c-Jun N-terminal kinase (JNK) signal pathway (Lu et al., 2012). Yasuzaki et al. (2013) has demonstrated that apelin/APJ signaling may promote Fas-induced liver injury via the phosphorylation of JNK in mice administered by intraperitoneal injection of an agonistic anti-Fas antibody (Jo2) (Yasuzaki et al., 2013). It was also shown that after partial hepatectomy and being given exogenous F13A, which is a specific APJ antagonist, liver regeneration was increased by enhancement of STAT3 and ERK1/2, but not phosphatidylinositol 3-kinase (PI3K)/Akt signaling. These results suggest that the apelin/APJ inhibits hepatocyte proliferation after partial hepatectomy in mice. Also, apelin was shown to possibly inhibit cell apoptosis in osteoblasts. Anti-apoptosis is mediated by the induction of Bcl2 protein expression and activation of the PI3K/Akt signaling pathway (Xie et al., 2007; Alastalo et al., 2011) (**Figure 1**).

The studies have reported that insulin, TNF-α, all-trans-retinoic acid (ATRA) and hypoxic conditions may increase apelin expression *in vivo* and *in vitro*, and some transcription factors may regulate apelin gene transcription or expression. However, in different tissues or cells, apelin/APJ expression has different levels with the same stimulant. For example, Daviaud et al. (2006) reported that TNF-α up-regulated apelin expression in human and mouse adipose tissue while Melgar-Lesmes et al. (2010, 2011) revealed that, in HSCs, TNF-α decreased apelin/APJ expression at mRNA and protein levels, but increased apelin/APJ expression in HepG2 cells. Overexpression of Stat3 and upstream stimulatory factor 1/2 may increase apelin promoter transcription activity (Wang et al., 2006; Han et al., 2008). Increased apelin/APJ in liver tissues has been shown to occur with hypoxia (Melgar-Lesmes et al., 2011). Under hypoxic conditions, hypoxia inducible factor-1α (HIF-1α) binds to the first intron of the apelin gene and the hypoxia-responsive element in the apelin promoter. It then increases apelin expression (Ronkainen et al., 2007; Eyries et al., 2008). The studies have identified that apelin promoter transcriptional activity increased through endothelial myocyte enhancer factor 2 (MEF2) transcription factors in the developing cardiovascular system (Kang et al., 2013), but its activity was inhibited by ATF4 in HepG2 cells (Jeong et al., 2014). The molecular mechanisms of the regulation of APJ gene transcription, however, are still unknown. The other investigations have also shown that both APJ and apelin genes are probably regulated by specificity protein 1 (Sp1) (O’Carroll et al., 2006; Hata et al., 2007). Our previous study showed that Sp1 and RARα formed a transcriptional activation complex, leading to an up-regulation of apelin expression in rat VSMCs (Lv et al., 2013).

## Function of the Apelin/APJ in Physiology and Pathophysiology of the Liver

In the liver, the apelin/APJ system could play an inhibitory role in liver regeneration and promote Fas-induced apoptosis. It may participate in formation of hepatic fibrosis or cirrhosis, and even cancer. The expression of apelin/APJ and related liver disease has been shown in **Table 1**.

**Table 1:** Expression of the apelin/APJ system in patients, tumor tissues, or cell lines.

Liver disease type	Patients/tissues/cells	mRNA	Protein	Serum levels	Reference
Partial hepatectomy	Mouse kupffer cell	APJ↑	–	–	Yoshiya et al., 2015
Hepatocellular carcinoma	Liver of hepatocellular carcinoma patients	Apelin↑	–	–	Muto et al., 2014
Alcoholic liver cirrhosis	Human serum	–	–	Apelin↑	Kalafateli et al., 2015
Non-alcoholic fatty liver disease	Human serum	–	–	Apelin↑	Aktas et al., 2011
Liver cirrhosis	Human serum, Rat and human tissues	Apelin↑ APJ↑	Apelin↑ APJ↑	Apelin↑	Principe et al., 2008; Melgar-Lesmes et al., 2010, 2011; Yokomori et al., 2011; Lim et al., 2016
Fas-induced liver injury	Mouse tissues	APJ↑	APJ↑	–	Yasuzaki et al., 2013
Hepatitis C	Patients HSCs, hepatocytes	Apelin↑	Apelin↑	Apelin↑	El-Mesallamy et al., 2011; Farid et al., 2014

Apelin/APJ signaling may promote Fas-induced liver injury via JNK activation. In the APJ^-/-^ mice, it had been demonstrated that liver apoptosis and injury were significantly alleviated compared with that in wild-type mice, and JNK activation was strongly inhibited (Yasuzaki et al., 2013). F13A, a specific APJ antagonist, could directly promote the activation of Kupffer cells and enhance the secretion of TNF-α and IL-6, which could increase hepatocyte proliferation with partial resection of the liver. However, this effect has not arisen in hepatocytes or hepatic stellate cells. Moreover, the evidence revealed that the relationship between apelin and TNF-α was important in the development of liver regeneration (Fausto et al., 2012). Sagiroglu et al. (2014) reported that exogenous apelin administration as pharmacological preconditioning alleviated hepatic ischemia reperfusion (I/R) injury in rats. These findings suggest that apelin/APJ system is involved in the process of liver regeneration.

The study showed that apelin was associated with the development of liver fibrosis. The *in vivo* study revealed that apelin/APJ could accelerate fibrosis progression in CCL_4_-treated rats (Reichenbach et al., 2012). Melgar-Lesmes et al. (2011) reported that apelin expression in HSCs was enhanced under hypoxic or proinflammatory conditions, and apelin could increase the synthesis of platelet-derived growth factor β receptor (PDGF-β) and collagen-I in LX-2 cells. Yokomori et al. (2011) reported that, in cultured HSCs, APJ expression was sharply increased by PDGF-β. The function of apelin/APJ in liver fibrosis still needs to be investigated.

A recent clinical investigation reported that serum apelin was associated with histological and hemodynamic states of chronic liver disease (Lim et al., 2016). Drougard et al. (2014) reported that hypothalamic apelin regulated hepatic glucose metabolism in mice fed a high-fat diet. Moreover, in human NALD, plasma apelin-12 levels were higher than that in healthy individuals (Ercin et al., 2010). The levels of apelin were also positively correlated with the homeostasis model assessment (HOMA) index and body mass index (BMI) in liver metabolic disease.

Recent studies have also reported that the apelin/APJ system participates in many kinds of cancer including lung, gastroesophageal, colonic, prostate and endometrial cancer (Picault et al., 2014; Altinkaya et al., 2015; Lv et al., 2016). The functions of apelin in liver cancer are still not clear. Apelin expression was upregulated in F26/KMUH cancer-associated fibroblast (CAFs) in HCC. Also, the higher levels of apelin/APJ expression were found in HCC based on clinical specimens. Tumor growth was obviously inhibited after blocking apelin/APJ signaling with exogenous F13A in a HCC subcutaneous mouse tumor model, compared to the control group (Muto et al., 2014). These results suggest that apelin/APJ is associated with liver cancer but the roles of apelin/APJ in liver cancer need to be further investigated.

## Conclusion

Recent studies have reported there are multiple roles for the apelin/APJ system in liver disease, including acute liver injury, liver regeneration, formation of cirrhotic liver and fibrosis progression. Apelin/APJ has unique functions as a regulator of cell proliferation, apoptosis, pro-inflammatory activity and revascularization. In addition, apelin/APJ gene expression is temporally increased during liver cirrhotic development and it decreased in stabilized liver fibrosis formation. The validation of using apelin/APJ as a biomarker in different liver diseases would also be a crucial step toward its clinical use. Also, further experimental or clinical findings will help to determine the potential of therapeutic strategies targeting the apelin/APJ system for treatment of liver disease.

## Author Contributions

XL wrote the manuscript. JK helped to prepare figures. W-DC and Y-DW reviewed, edited and revised the manuscript.

## Conflict of Interest Statement

The authors declare that the research was conducted in the absence of any commercial or financial relationships that could be construed as a potential conflict of interest.
